# Effect of Cerium Oxide Nanoparticles on Oxidative Stress Biomarkers in Rats’ Kidney, Lung, and Serum

**DOI:** 10.29252/ibj.24.4.251

**Published:** 2020-01-10

**Authors:** Adel Sepanjnia, Hassan Ghasemi, Roohollah Mohseni, Akram Ranjbar, Fatemeh Shabani, Fouzieh Salimi, Nejat Kheiripour

**Affiliations:** 1 Department of Biomedical Science, School of Medicine, Jiroft University of Medical Sciences, Jiroft, Iran;; 2 Department of Clinical Biochemistry, Abadan Faculty of Medical Sciences, Abadan, Iran;; 3 Department of Biochemistry, Faculty of Medicine, Shahrekord University of Medical Sciences, Shahrekord, Iran;; 4 Department of Toxicology and Pharmacology, School of Pharmacy, Hamadan University of Medical Sciences, Hamadan, Iran;; 5 Research Center for Biochemistry and Nutrition in Metabolic Diseases, Kashan University of Medical Sciences, Kashan, Iran

**Keywords:** Kidney, Lung, Nanoparticles, Oxidative stress

## Abstract

**Background:**

The present study aimed to evaluate the effects of different concentrations of CONPs on the OS status in kidney, lung, and serum of rats.

**Methods:**

Male Wistar Rats were treated intraperitoneally with 15, 30, and 60 mg/kg/day of CONPs. The biochemical parameters, including TAC, TTG, MDA, SOD, and CAT were assayed in serum, kidney, and lung tissues.

**Results:**

MDA decreased, but TTG and CAT increased in serum by the administration of CONPs at 15 mg/kg. In kidney homogenate obtained from the group treated with CONPs at 15 mg/kg, TAC, TTG, and CAT significantly increased compared to the control group. However, CONPs at 15, 30, and 60 mg/kg significantly decreased MDA level compared to the control group. In lung tissue, CONPs in doses of 15, 30 and 60 mg/kg significantly decreased CAT activity, TTG and TAC compared to the control group, while in kidney tissue, CONPs at the concentrations of 30 and 60 mg/kg significantly increased MDA compared to the control group.

**Conclusion:**

Our findings suggest that CONPs attenuate OS in the kidney and affect the serum levels of OS-related markers but induce OS in the lung tissue in a dose-dependent manner.

## INTRODUCTION

In the last decades, nanotechnology has developed a novel approach to the treatment and improvement of many diseases by the reduction of OS. Several nanoparticles such as CONPs have been designed for this reason^[^^1^^]^. Cerium, as a lanthanide, has a variety of industrial applications and has recently been used in nanomedicine research. CONPs consist of a cerium core that is surrounded by an oxygen lattice. It is widely employed in ultraviolet absorbents, solar cells, solid fuel cells, and so on^[^^2^^,^^3^^]^. 

OS means an imbalance between the production and degradation of free radicals and plays an important role in inflammation and tissue damage. The reduction of OS by increasing antioxidant capacity has been the best way for the improvement of related disorders^[^^4^^]^. CONPs have been reported to reduce OS and could scavenge ROS *in vitro* and *in vivo*^[^^5^^]^. It has also been shown that CONPs prevent OS injury in endothelial cells and reduce necrosis and apoptosis in response to ROS^[^^6^^]^. CONPs are able to control the cardiac, and kidney damage is induced by OS^[^^7^^,^^8^^]^. Guo *et al.*^[^^9^^]^ have demonstrated the protective effect of CONPs against OS by modulating TGF-beta signaling. 

Although many different studies mentioned above have confirmed the antioxidant properties of CONPs, some others have revealed that CONPs may induce OS and tissue damage in high concentrations and low pH^[^^10^^]^. Besides, studies have indicated that CONPs produce significant OS in the lung cancer cells via the reduction of glutathione and alpha-tocopherol^[^^11^^]^. CONPs can mediate apoptosis and DNA damage through OS in human skin melanoma cells and induce OS through the p38-Nrf2 signaling pathway in the human bronchial epithelial cell^[^^12^^]^.

Given the conflicting roles of CONPs, the current study was designed to assess the effect of different concentrations of CONPs on OS status in serum, lung, and kidney of male rats. We also determined the effect of CONPs on OS markers, including SOD and CAT activity, MDA, TAC and TTG, in serum, lung, and kidney. 

## MATERIALS AND METHODS

**Reagents and chemicals**

Reagents and materials used in this study include Ethylene-diamine-tetra-acetic acid, Coomassie Blue, BSA, 2,4,6-Tripyridyl-s-triazine, DTNB, Tris base, hydrochloric acid, ferric chloride, and ferrous sulfate that all were obtained from the Sigma Chemical Co. (USA). The CONPs (100 nm) were purchased from the Neutrino Co. (Iran). The nanoparticles were suspended in deionized water. SOD and CAT assay kits were supplied from ZellBio GmbH (Ulm, Germany). All the other chemicals used were of the analytical grade. 

**Animals’ treatment**

In total, 20 male Wistar rats (weight: 220 ± 20 g) were obtained from the Animal Colony of Hamadan University of Medical Sciences, Hamadan, Iran. The animals were preserved in standard conditions with a temperature of 22 ± 1 ºC, humidity of 45-55%, and 12-hour light/dark cycle. The rats were ‎randomly divided into four groups (five animals per group). Group 1 included healthy controls received normal saline and groups 2, 3, and 4 received CONPs 15, 30, and 60 mg/kg/day intraperitoneally, respectively and continued for seven consecutive days. At the next stage, 24 hours after the last injection, the fasting rats were anesthetized with ketamine (50 mg/kg), and serum, kidney and lung samples were then collected. 

**Serum and tissue perpetration**

Blood samples were collected from the heart, and serum was isolated quickly and kept at -20 °C. Also, kidney and lung tissues were excised and collected from all groups immediately. Tissues were then homogenized (10 mg of tissue in 140 mM of cold phosphate buffer saline, pH 7.4). The homogenate was centrifuged at 10,000 ×g at 4 °C for 15 minutes, and the supernatant was collected and maintained at -80 °C.

**Biochemical analysis**

***Assay of OS parameters***

OS parameters were assayed by the ferric reducing ability of plasma method. This approach is based on the plasma ability to reduce Fe^3+^ to Fe^2+^. The reaction of Fe^2+^ and 2,4,6-Tripyridyl-s-triazine produces a blue complex with maximum absorbance at 593 nm^[^^13^^]^. To evaluate the plasma TTG, DTNB was used as a reagent. DTNB reacts with thiol molecules and creates a yellow complex, which has appropriate absorbance at 412 nm in spectro-photometer^[^^14^^]^. MDA, a marker of lipid peroxidation, was measured by using the colorimetric method, which is based on a peroxidized lipid reaction with thiobarbituric acid. The reaction product was measured by using 1,1,3,3-Tetraethoxy-propane standard curve in 532 nm^[^^15^^]^.

***Assessment of antioxidant enzymes activity***

CAT activity was measured using a calorimetrically enzymatic assay kit at 405 nm (ZellBio GmbH, Ulm, Germany). In this assay, the CAT activity unit was considered as the amount of the sample that will catalyze decomposition of 1 µmole of H_2_O_2_ to H_2_O and O_2_ in 1 minute. This method can determine CAT with 0.5 U/mL of sensitivity. The intra- and inter-assay coefficient of variation was claimed to be 6.3% and 7.9%, respectively. SOD activity was measured using a calorimetrically enzymatic assay kit (ZellBio GmbH, Ulm, Germany). In this assay, the SOD activity unit was considered as the amount of the sample that will catalyze the decomposition of 1 mmol of O_2_ to H_2_O_2_ and O_2_ in 1 minute. The SOD activity was determined colorimetrically at 420 nm.

***Measurement of total protein***

Protein concentration in the samples was measured by the Bradford method using concentrated Coomassie blue reagent. Also, BSA was used as a standard^[^^13^^]^.

**Statistical analysis**

All data were expressed as mean ± SD. The results were analyzed by SPSS 16. Statistical analysis was performed using one-way analysis of variance (ANOVA), followed by post hoc Tukey’s test. *p* < 0.05 was considered statistically as significant level.

**Ethical statement**

The above-mentioned sampling protocols were approved by the Medical Ethics Review Board of Jiroft University of Medical Sciences, Kerman (ethical code: IR.JMU.REC.1393.28).

**Fig. 1:**
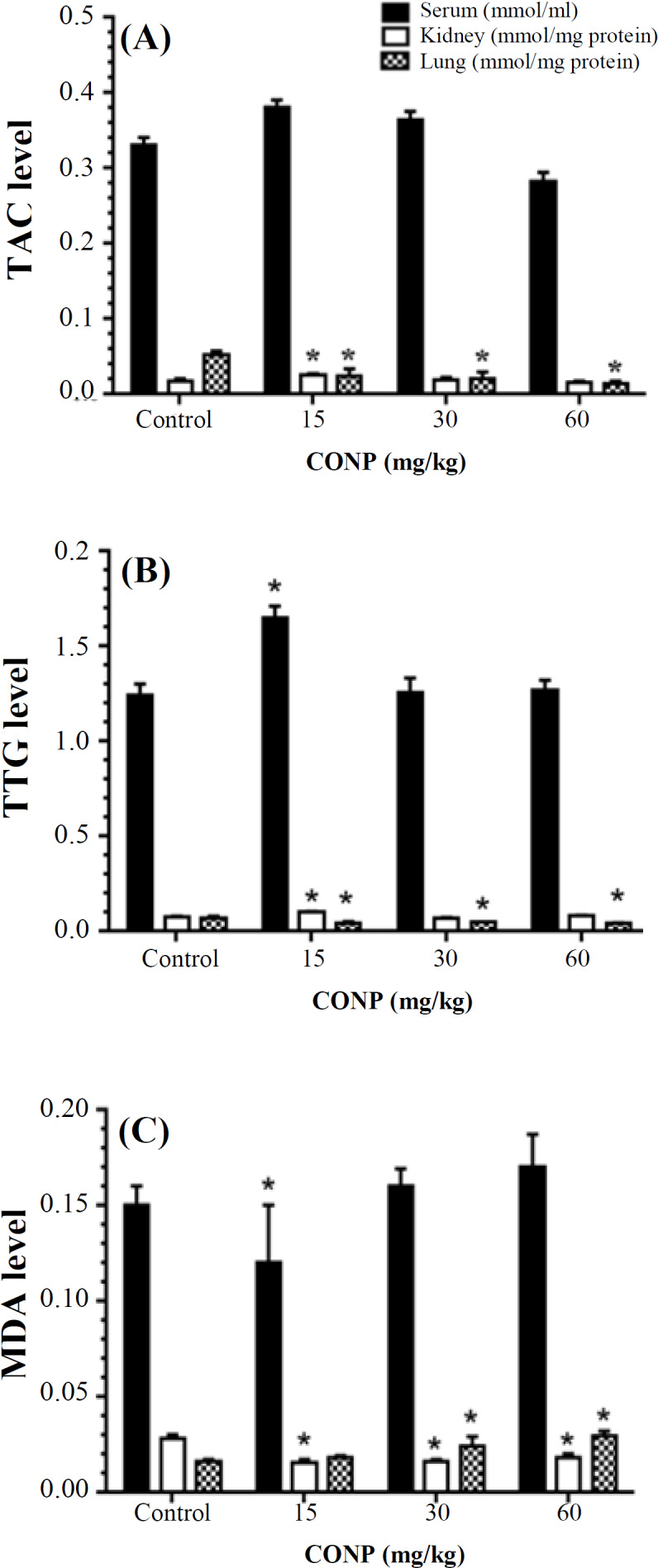
Effect of CONPs treatment on TAC, TTG and MDA level in serum, kidney, and lung. Results are presented as means ± SD. CONPs in doses of 15 mg/kg showed a significant increase in TAC level in kidney, and TTG level in serum and kidney, but in the CONPs 15, 30, and 60 mg/kg group, CONPs therapy indicated a significant decrease in TAC and TTG level in lung tissue, as compared with the control group (^*^p < 0.05). CONPs therapy showed a significant decrease in MDA level in serum (15 mg/kg) and kidney (15, 30 and 60 mg/kg) tissue compared with the control group. In the CONPs 30 and 60 mg/kg group, CONPs therapy showed a significant increase in MDA level in lung tissue, as compared with the control group (*p < 0.05)

## RESULTS

The experimental models received different doses of CONPs (15, 30, and 60 mg/kg) and OS biomarkers (TAC, TTG, and MDA levels), and CAT and SOD activity in serum, kidney, and lung were measured. All experiments repeated at least three times.

***OS parameters***

Serum TAC levels (Fig. 1A) showed no significant difference between all the groups (*p* > 0.05). CONPs at 15 mg/kg caused a significant increase in the TAC level in kidney, but at doses of 15, 30, and 60 mg/kg, it decreased lung TAC level significantly, when compared to the control group (*p* < 0.05). The serum and kidney TTG levels in the treatment group receiving 15 mg/kg of CONPs were higher than the control rats (*p* < 0.05). At doses of 15, 30, and 60 mg/kg, CONPs suppressed the TTG level in the lung compared with the normal groups (Fig. 1B). Based on the Figure 1C, treatment with CONPs (15 mg/kg) resulted in a significant decrease in serum MDA level compared to the control group. In kidney tissue, the MDA level of CONPs treated with three dose groups was significantly reduced compared to the control group (*p* < 0.05). However, in the lung tissue, CONPs at doses of 30 and 60 mg/kg significantly increased the MDA level compared with the normal rats (*p* < 0.05).

***Antioxidant enzyme activity***

**Fig. 2:**
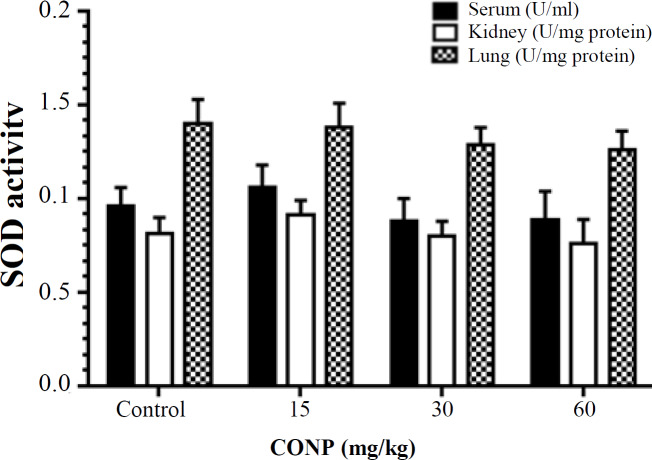
Effect of CONPs treatment on SOD level in serum, kidney, and lung. Results are presented as means ± SD. CONPs (15, 30 and 60 mg/kg) therapy showed a non-significant effect on SOD activity in serum, kidney, and lung tissue, as compared with the control group (^*^p < 0.05)

**Fig. 3:**
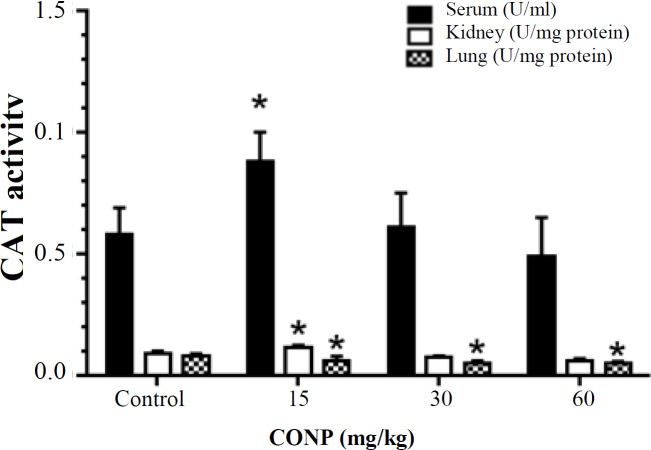
Effect of CONPs treatment on CAT activity in serum, kidney, and lung. Results are presented as means ± SD. In the CONPs 15 mg/kg group, CONPs therapy showed a significant increase in CAT activity in serum and kidney but in the CONPs 15, 30, and 60 mg/kg group, CONPs therapy indicated a significant decrease in CAT activity in lung tissue, as compared with the control group (^*^p < 0.05).

According to the observations, the level of SOD activity between the studied groups showed no significant difference (*p* > 0.05; Fig. 2). Also, according to the results presented in Figure 3, serum and kidney CAT activity in the CONPs at 15 mg/kg group significantly increased (*p* < 0.05) compared to the control groups. However, in the lung, CAT activity in all the groups treated with CONPs significantly decreased compared to the control rats (*p* < 0.05).

## DISCUSSION

Metal oxide nanoparticles such as CONPs play a very important role in reducing OS that occurs in various diseases^[^^16^^,^^17^^]^. CONPs are one of the most popular nanoparticles that scavenge free radicals. A previous study has reported that treatment with CONPs could reduce OS status in the tissue and serum^[^^18^^]^.

Although there are many various studies confirming CONPs antioxidant properties, others have suggested that CONPs may increase OS and damage tissue, such as lung and liver, in high concentration and low pH^[^^12^^,^^18^^]^. Because of the high vascularity and the possibility of nanoparticle accumulation in the lung and kidney, in this study, we decided to analyze the effect of CONPs treatment on OS factors, including SOD and CAT activity, MDA, TAC, and TTG concentration in lung, kidney, and serum.

Our results showed that CONPs treatment increased TAC in kidney just with a dose of 15 mg/kg against the control group, significantly. Also, nanoparticle treatment significantly increased TTG in 15 mg/kg both in kidney and serum. In contrast, treatment with CONPs decreased TAC and TTG levels in lung tissue. These results support the previous evidence that disclosed CONPs increased total thiol and total antioxidant power in kidney, heart, and brain tissues but decreased in lung in experimental diabetic model^[^^19^^]^. 

In this study, CONPs decreased lipid peroxidation in kidney and serum, especially with a 15 mg/kg dose. However, treatment with CONPs resulted in the increased lipid peroxidation level in lung tissue in a dose-dependent manner. Therefore, nanoparticle exposure may lead to tissue damage through ROS production in the lung. Eom and Choi^[^^12^^]^ have disclosed that CONPs induce OS in bronchioles cells via increasing free radicals.

According to our findings, CONP treatment did not have any effect on SOD activity in tissue and serum. These observations do not support the previous evidence that treatment with CONPs protect gastrointestinal epithelial damage against radiation through SOD production^[^^20^^]^. Nanoparticle exposure significantly elevated CAT activity in kidney and serum by administration of only 15 mg/kg but decreased CAT activity in lung, similar to other antioxidant parameters such as TAC and TTG. Earlier studies have demonstrated that CONPs reduce inflammation and ROS production and maintain enzymatic antioxidants and significantly reduce lipid peroxidation in the kidney^[21,22]^. According to a number of studies, CONPs have CAT mimetic activity that may be responsible for increasing CAT activity in the present study^[^^22^^,^^23^^]^. 

The current research revealed that the antioxidant effect of nanoparticle in the kidney and serum was dose-dependent in the rat. CONPs exert a destructive effect on the lung tissue and cause OS. Antioxidant effect of CONPs in serum and kidney has been approved by Chen *et al.*^[^^6^^] ^who showed CONPs prevented OS injury in endothelial cells. Pagliari *et al.*^[^^25^^]^ have also exhibited that CONPs reduce ROS-induced cell damage in cardiac progenitor cells. CONPs decrease ROS level and cell damage in smokers through NF-κB activation, regulation of inflammatory genes expression, and antioxidant depletion^[^^26^^]^. In addition, Guo *et al.*^[^^9^^]^ demonstrated that CONPs have OS protection property by the modulation of TGF-beta signaling.

Experimental data from lung tissue have been confirmed by recent findings. Eom and Choi^[^^12^^]^ have shown that CONPs produce OS in human epithelial cells through p38-Nrf-2 signaling pathway. In addition, CONPs can mediate apoptosis and DNA damage by increasing OS in human skin melanoma cells^[^^2^^]^. CONPs produce OS in the cells, as reflected by reduced glutathione and alpha-tocopherol levels in human lung cancer cells^[^^11^^]^.

In summary, the findings of the present study demonstrate that CONPs may attenuate intracellular OS and increase enzymatic antioxidant activity in serum and kidney in a dose-dependent manner. However, the exposure of nanoparticle in lung induces ROS production and decreases antioxidant factors. More study is needed to determine the exact molecular mechanism of these events.
